# Effects of Yi Jin Jing on enhancing muscle strength and physical performance in older individuals: a systematic review and meta-analysis

**DOI:** 10.3389/fmed.2024.1441858

**Published:** 2024-10-25

**Authors:** Xiaoping Zhang, Wenda Jiang, Zhenqi Chen, Guang Yang, Zhongyu Ren

**Affiliations:** ^1^College of Physical Education, Chinese Center of Exercise Epidemiology, Northeast Normal University, Changchun, Jilin, China; ^2^College of Physical Education, Jilin Normal University, Siping, Jilin, China; ^3^College of General Education, Guangxi Arts University, Nanning, Guangxi, China; ^4^College of Physical Education, Chinese Center of Exercise Epidemiology, Southwest University, Chongqing, China

**Keywords:** Yi Jin Jing, traditional Chinese exercise, older individuals, muscle strength, physical performance

## Abstract

**Background:**

The aging population is rapidly increasing, leading to physical decline and higher risks of chronic diseases, including sarcopenia, which adversely affects muscle quality and strength. Yi Jin Jing (YJJ), a traditional Chinese exercise method, can enhance flexibility and strength, but evidence regarding its effectiveness in older adults is conflicting. This meta-analysis aims to systematically evaluate the effects of YJJ on muscle strength and physical performance in this demographic.

**Methods:**

We searched seven electronic databases: China National Knowledge Infrastructure, Wanfang Data, Sinomed, Web of Science, PubMed, Cochrane Library, and EMBASE to identify randomized controlled trials (RCTs). Following PRISMA guidelines, we quantified the effects of YJJ on muscle strength (grip strength, isokinetic strength) and physical performance (chair sit-to-stand, squatting-to-standing, shoulder flexibility, sit-and-reach tests). Treatment effects were calculated using Hedges’g. The Cochrane tool assessed risk of bias, the PEDro scale evaluated methodological quality, and the GRADE method assessed evidence quality. Data analysis was conducted using Stata 17.0 software, utilizing standardized mean differences (SMD) and 95% confidence intervals (CIs).

**Results:**

This meta-analysis included 10 RCTs involving 590 participants. The overall risk of bias was assessed to be low. The methodological quality of these studies was generally moderate, and the quality of the main results varied from low to moderate. The findings revealed that YJJ had considerable effects on the chair sit-to-stand test (Hedges’g = 1.06), squatting-to-standing test (Hedges’g = 1.08), and small to moderate effects on handgrip strength (Hedges’g = 0.25), 60°/s extensor peak torque (Hedges’g = 0.47), 60°/s extensor average power (Hedges’g = 0.31), 60°/s extensor total work (Hedges’g = 0.29), 60°/s flexor peak torque (Hedges’g = 0.42), 60°/s flexor average power (Hedges’g = 0.37), and 180°/s extensor peak torque (Hedges’g = 0.29), and left shoulder flexibility (Hedges’g = 0.4). However, there were no significant improvement effects in 180°/s extensor average power (Hedges’g = 0.19), 180°/s extensor total work (Hedges’g = 0.11), 180°/s flexor peak torque (Hedges’g = 0.01), 180°/s flexor average power (Hedges’g = −0.08), right shoulder flexibility (Hedges’g = 0.09), and sit-and-reach test (Hedges’g = 0.15).

**Conclusion:**

YJJ significantly enhances specific aspects of physical performance, particularly chair sit-to-stand and squatting-to-standing tests, while showing small and moderate improvements in handgrip strength and knee muscle strength. However, it had no significant effects on other metrics, including shoulder flexibility and sit-and-reach tests.

**Systematic review registration:**

https://www.crd.york.ac.uk/prospero/display_record.php?ID=CRD42024530487, Registration number: CRD42024530487.

## 1 Introduction

China’s population is experiencing an accelerated aging process, characterized by a noticeable shift toward older age groups. A national survey conducted in 2021 revealed that the older population in China constitutes 18.7% of the total population, with a continuing upward trend (1). Aging is associated with a decline in physiological ability, rendering individuals highly susceptible to various chronic diseases, including diabetes (2), hypertension (3), and Parkinson’s disease (4). Notably, a recent comprehensive meta-analysis and systematic review reported that the prevalence of sarcopenia in the older Asian population was 21.6%, significantly higher than that in other regions (5). Sarcopenia is defined as a significant loss of muscle mass and strength associated with aging, disease, or other contributing factors (6). This condition adversely affects individual health and increases the risk of falls (7), fractures (8), and mortality (9). Furthermore, these consequences substantially decrease the quality of life and impose a heavy burden on healthcare systems (10).

The implementation of preventive measures is essential to reduce the incidence of age-related diseases. The Asian Working Group for Sarcopenia (AWGS) 2019 introduced the concept of “possible sarcopenia,” which is defined as either low muscle strength or low physical performance only (11). This definition aims to aid early interventions in primary healthcare environments and community initiatives. Therefore, it is imperative to promptly identify and implement effective prevention strategies to halt the progression of sarcopenia in the older individuals.

Yi Jin Jing (YJJ) is a traditional Chinese exercise method that involves slow, controlled movements aimed at improving flexibility, strength, and overall physical performance (12). The traditional YJJ comprises a standardized set of 12 movements, such as stretching, twisting, and squatting, which have gained widespread attention for their potential benefits in enhancing muscle strength and physical performance in older adults (13). Although YJJ is typically practiced in a single, consistent style, the movements emphasize controlled body mechanics and promote gradual improvements in physical capabilities (14). Based on a review of existing studies, there is conflicting evidence regarding the effects of YJJ on muscle strength and physical performance in older individuals (15–23). Although numerous studies have reported significant benefits (15–20), others have not (21–23). We further found that the diversity of research methodologies, participants’ characteristics, and exercise programs among the studies contributed to outcome variability. Additionally, the different definitions of sarcopenia and the measurement methods for physical performance also intensify the uncertainty regarding YJJ’s effectiveness.

Previous meta-analyses have shown that traditional Chinese exercises can improve physical health (24–27). However, these studies often combined various types of traditional Chinese exercises and failed to use Hedges’g statistics to estimate the effect size. The use of Hedges’g is particularly important given the small sample sizes in the analyzed studies, as it provides a more precise estimation, thereby improving the reliability of the findings (28). Therefore, this study aims to accurately calculate the effect size of YJJ on muscle strength and physical performance in older individuals by synthesizing existing studies. It addresses critical gaps in the literature by clarifying the inconsistencies caused by participant characteristics and the diversity of exercise programs. By using Hedges’g statistics, we improve the precision of effect size estimations, thereby improving the reliability of understanding the effectiveness of YJJ. The findings of this study are essential for guiding future research and developing practical interventions to enhance the well-being of the older individuals.

## 2 Method

The meta-analysis adhered to the Preferred Reporting Items for Systematic Reviews and Meta-Analyses (PRISMA) (29). This study was registered in the Prospective Register of Systematic Reviews (PROSPERO);^1^ registration number CRD42024530487.

### 2.1 Search strategy

From November 2023, a systematic search was conducted across seven electronic databases: CNKI, Wanfang Data, Sinomed, Web of Science, PubMed, Cochrane Library, and Embase. Additionally, manually search the reference list of relevant publications to find the non-indexed studies in these databases. The search terms used to search for relevant literature include the following combinations: (“Qigong” or “Chinese traditional exercise” or “Yijinjing” or “Yi jinjing” or “Yi Jin Jing”) AND (“muscle strength” OR “muscle strength” OR “grip” OR “physical activit*” OR “physical performance” OR “physical fitness” OR “motor function”) AND (“randomized controlled trial*” OR “random*” OR “clinical trial*”). The search strategies for different databases were modified accordingly. The final search date was March 2024.

### 2.2 Selection criteria

The selection criteria were as following:

- Study Type: The studies included were randomized controlled trials (RCTs). These were original, peer-reviewed works with no restrictions on language or publication date.
- Population: Participants were required to be older than 60 years and inclusion was not limited by sex, race, or country of origin.
- Intervention: The intervention had to exclusively involve YJJ and could not be combined with other exercise interventions. There were no specified limits on the frequency, intensity, or duration of YJJ.
- Comparator: The comparator group either received no intervention or was provided health education.
- Outcome Measures: Muscle strength measures included handgrip strength and isokinetic muscle strength. Physical performance assessments comprised chair sit-to-stand, squatting-to-standing, shoulder flexibility, and sit-and-reach tests.

### 2.3 Data management and extraction

Data were merged using EndNote, and duplicate entries were removed. Two independent reviewers assessed titles, abstracts, and full texts for eligibility. Any conflicts or ambiguities were resolved through discussion or arbitration with a third reviewer.

The two reviewers extracted relevant data, summarized them in Microsoft Excel spreadsheets, and cross-checked the results. The data extracted from the literature included the first author, publication date, participant age, sample size, sex composition, physical condition, diagnostic criteria, intervention type, intervention frequency and duration, measurement tools, and outcome indicators.

### 2.4 Quality assessment

The PEDro scale consists of 11 items to assess the methodological quality of the included study (30), with scores downloaded from PEDro (pedro.org.au). The first item (qualification criterion) was excluded from the total score because it did not directly reflect the internal validity of the study. Therefore, the score ranged from 0 (low quality) to 10 (high quality) and was divided into four quality levels: excellent (9–10 points), good (6–8 points), fair (4–5 points), and poor (0–3 points) (31).

The risk of bias for each study was assessed using the Cochrane Collaboration tool (32). According to the design and implementation of the original studies, three bias risk levels (high, low, and unclear) were determined for each item. The evaluation was conducted independently by two authors. Any differences were reviewed by a third evaluator and resolved by consensus.

### 2.5 Statistical analysis

The meta-analysis was performed using stata17.0. For continuous results, Hedges’g was used to calculate the between-groups effect sizes, which were classified as small (0.2–0.4), medium (0.5–0.7), or large (≥0.8) (33). The GRADE method was used to determine the certainty of the evidence (34). When there was a high degree of heterogeneity (*p* < 0.1 or *I*^2^ > 50%), a random-effects model was employed for meta-analysis, and the source of heterogeneity was identified through subgroup analysis or a stepwise method (35). Statistical significance was set at *p* < 0.05.

## 3 Results

### 3.1 Search results

**Figure 1:**
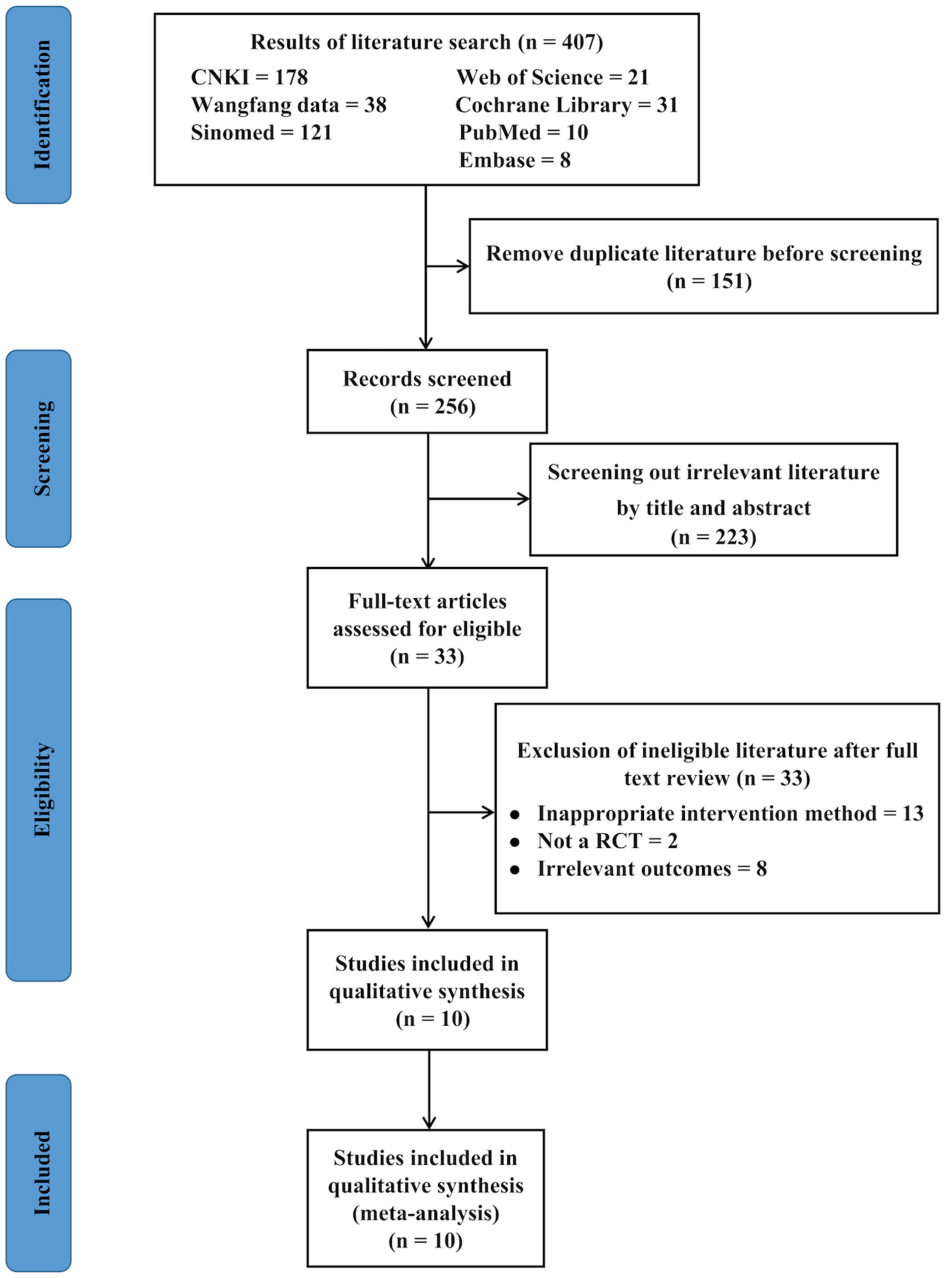
Study literature selection process flowchart.

Figure 1 summarizes the screening process. Our initial search yielded 407 articles, of which 151 were duplicates and were excluded. Subsequent screening of titles and abstracts resulted in the exclusion of an additional 223 items. Finally, 33 articles were thoroughly reviewed, 10 of which were included in this meta-analysis (15, 16, 18, 21–23, 36–39).

### 3.2 Study characteristics

Table 1 shows the characteristics of the studies included in this meta-analysis. However, there were 590 participants, all of whom were over 60 years old, and 75% were female. Two trials included only women (22, 23), one trial included only men (16), and the remaining included a mix of sexes (15, 18, 21, 36–39). Among the participants included in the study, one study focused on patients with knee osteoarthritis (36), two studies identified sarcopenia using the AWGS diagnostic method (15, 16), five studies identified sarcopenia according to Roubenoff’s view (18, 21, 37–39), and the other two studies involved healthy older individuals (22, 23). All experimental groups were treated with YJJ, and the control group received either health education (15, 39) or a blank control (16, 18, 21–23, 36–38). The results of these 10 studies were as follows: handgrip strength (15, 16, 21–23); 60°/s isokinetic muscle strength (18, 36, 38, 39), 180°/s isokinetic muscle strength (18, 36, 38, 39), chair sit-to-stand test (15, 21, 37), squatting-to-standing test (15, 21, 37), shoulder flexibility test (21, 37), and sit-and-reach test (21, 22, 37).

**Table 1:** Characteristics of each study included in the meta-analysis.

Number	Study	Health condition	Diagnostic criteria	Trial group *vs* control group			Exercise frequency and duration	Outcomes
				Average age	Sample size (male/female)	Interventions		
1	Fan et al. 2012	Knee Osteoarthritic	/	60.35 ± 0.53 61.21 ± 8.7	45 (5/40) 45 (9/36)	YJJ vs. None	NR min/session, 7 sessions/week for 8 weeks	②^†^ ③^†^
2	Gong et al. 2011	Sarcopenia	Roubenoff’s view	67.0 ± 5.28 66.4 ± 5.47	30 (9/21) 30 (7/23)	YJJ vs. None	20 min/session, 3 sessions/week for 8 weeks	②^†^ ③
3	Jin et al. 2011	Sarcopenia	Roubenoff’s view	68.22 ± 4.09 65.09 ± 3.95	36 (14/22) 35 (7/28)	YJJ vs. None	60 min/session, 3 sessions/week for 8 weeks	④^†^ ⑤^†^ ⑥ ⑦
4	Liu et al. 2016	Sarcopenia	Roubenoff’s view	67.86 ± 6.86 69.10 ± 6.69	31 (2/19) 30 (12/18)	YJJ vs. Health education	30 min/sessions, 3 sessions/week for 8 weeks	②^†^ ③^†^
5	Liu et al. 2010	Health	/	65.70 ± 3.10 65.70 ± 3.10	32 (0/32) 30 (0/32)	YJJ vs. None	40–50 min/sessions, 6 sessions/week for 24 weeks	①
6	Su et al. 2012	Health	/	61.40 ± 5.60 61.40 ± 5.60	35 (0/35) 35 (0/35)	YJJ vs. None	60 min/session, 5 sessions/week for 12 weeks	① ⑦^†^
7	Wang et al. 2016	Sarcopenia	Roubenoff’s view	66.79 ± 4.76 65.59 ± 3.59	38 (15/23) 37 (7/30)	YJJ vs. None	60 min/session, 3 sessions/week for 12 weeks	①^†^ ④^†^ ⑤^†^ ⑥^†^ ⑦
8	Wang et al. 2015	Sarcopenia	Roubenoff’s view	66.79 ± 4.76 65.59 ± 3.59	13 (7/6) 13 (3/10)	YJJ vs. None	60 min/session, 3 sessions/week for 12 weeks	②^†^ ③
9	Zhao et al. 2016	Sarcopenia	AWGS	67.80 ± 3.80 66.00 ± 3.1 L	6 (6/0) 6 (6/0)	YJJ vs. None	20 min/session, 3 sessions/week for 8 weeks	①^†^
10	Zhu et al. 2019	Sarcopenia	AWGS	66.30 ± 10.80 65.60 ± 11.40	32 (17/15) 31 (15/16)	YJJ vs. Health education	40 min/session, 7 sessions/week for 12 weeks	①^†^ ④^†^ ⑤^†^

① Handgrip strength; ② 60°/s isokinetic muscle strength; ③ 180°/s isokinetic muscle strength; ④ Chair sit-to-stand test; ⑤ Squatting-to-standing test; ⑥ Shoulder flexibility; ⑦ Sit-and-reach test; ^†^*p* < 0.05.

### 3.3 Quality of the evidence

**Figure 2:**
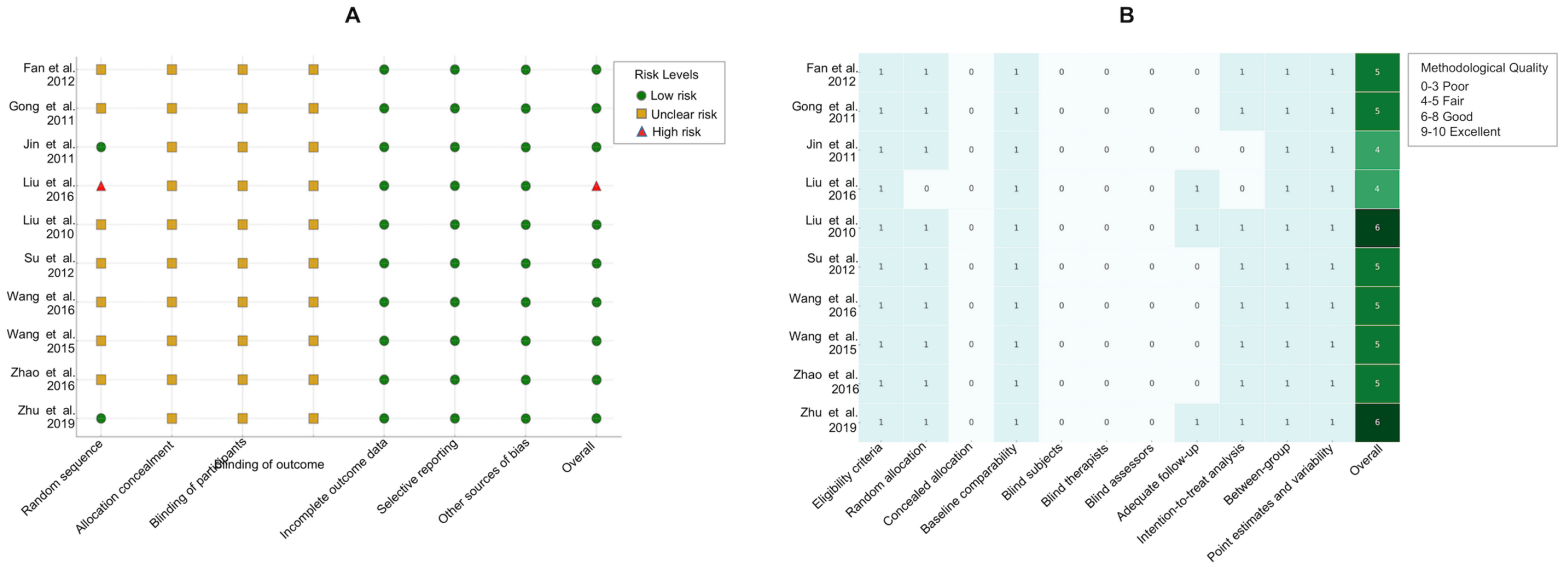
**(A)** Bias risk assessment and **(B)** methodological quality of including studies.

Figure 2A illustrates the results of bias risk assessment for the included studies. In the risk of bias assessment, one study (39) was classified as high risk owing to bias in the randomization process. Two studies (15, 37) used the random number table method for random allocation. None of the studies (15, 16, 18, 21–23, 36–39) did not mention allocation concealment or the use of blinding for evaluating results. Additionally, owing to the nature of the included studies, blinded participants and therapies were not applicable.

Figure 2B shows the methodological quality of the included studies assessed using the PEDro scale (31). Two studies (15, 23) were rated as “good” because they provided detailed records of dropout during the testing process or conducted measurements at multiple time points, while the remaining studies (16, 18, 21, 22, 36–39) were rated as “fair.”

### 3.4 Outcome measures

#### 3.4.1 Muscle strength in the older individuals

**Figure 3:**
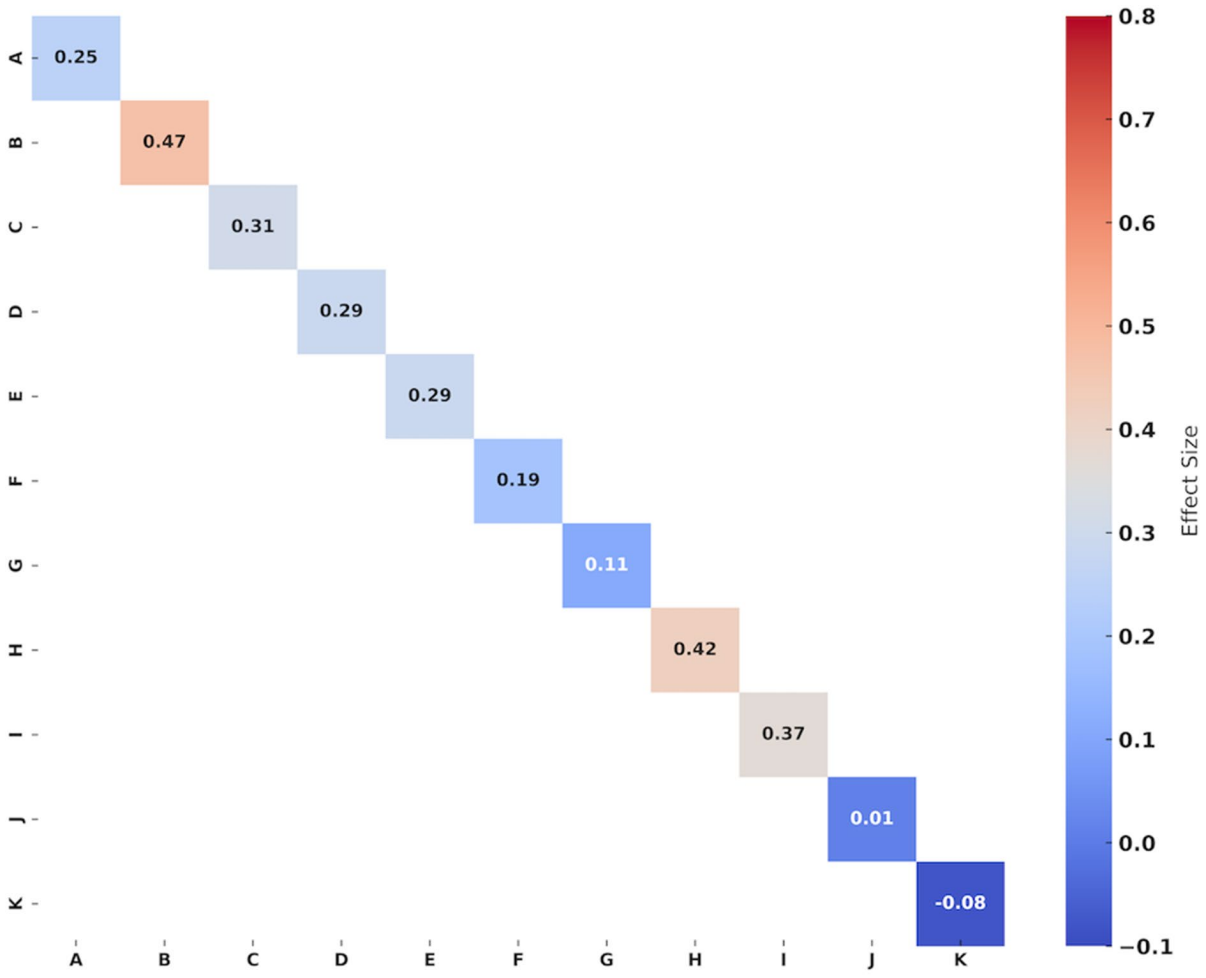
Effect size of Yi Jin Jing on muscle strength in the older individuals. A: Handgrip strength; B: 60°/s extensor’ PT; C: 60°/s extensor’ AP; D: 60°/s extensor’ TW; E: 180°/s extensor’ PT; F: 180°/s extensor’ AP; G: 180°/s extensor’ TW; H: 60°/s flexor’ PT; I: 60°/s flexor’ AP; J: 180°/s flexor’ PT; K: 180°/s flexor’ AP. Hedges’g values of 0.2–0.4 indicating small, 0.5–0.7 signifying medium, and ≥0.8 representing large effect sizes, respectively.

Table 2 and Figure 3 summaries the evidence for the effect of YJJ on muscle strength in the older population. Forest plots and specific grade ratings are provided in Supplementary material.

**Table 2:** Summary of evidence on the impact of YJJ on muscle strength in the older individuals.

Outcomes	Number of studies	Effect model	*I*^2^ (%)	Effect size (95%CI)	*p*-value	Certainty of evidence
Handgrip strength	5	Fixed model	0	1.18 (−0.22, 2.58)	0.03	⨁⨁◯◯ LOW
60°/s extensor’ PT	4	Fixed model	0	9.26 (4.58, 13.95)	<0.001	⨁⨁⨁◯ MODERATE
60°/s extensor’ AP	4	Fixed model	12.88	4.48 (1.15, 8.53)	0.02	⨁⨁⨁◯ MODERATE
60°/s extensor’ TW	3	Fixed model	8.79	76.21 (5.17, 147.26)	0.03	⨁⨁⨁◯ MODERATE
180°/s extensor’ PT	4	Fixed model	27.03	4.46 (1.02, 7.90)	0.02	⨁⨁⨁◯ MODERATE
180°/s extensor’ AP	4	Fixed model	19.98	5.25 (0.42, 10.91)	0.14	⨁⨁⨁◯ MODERATE
180°/s extensor’ TW	3	Fixed model	0	18.05 (20.11, 56.21)	0.41	⨁⨁⨁◯ MODERATE
60°/s flexor’ PT	3	Fixed model	19.21	5.56 (1.54, 9.77)	0.01	⨁⨁⨁◯ MODERATE
60°/s flexor’ AP	3	Fixed model	0	2.99 (0.38, 5.60)	0.02	⨁⨁⨁◯ MODERATE
180°/s flexor’ PT	3	Fixed model	0	−0.06 (−0.29, 2.78)	0.96	⨁⨁⨁◯ MODERATE
180°/s flexor’ AP	3	Fixed model	0	−0.17 L (−4.44 L, 4.09)	0.61	⨁⨁⨁◯ MODERATE

PT, peak torque; AP, average power; TW, total work.

Five studies (15, 16, 21–23) reported handgrip strength outcomes. The synthesized data indicated that compared to the control group, the effect of YJJ on enhancing handgrip strength in the older individuals was statistically significant (*I*^2^ = 0%, *p* = 0.03, certainty of evidence = low), with a small improvement effect (Hedges’g = 0.25).

In the assessment of lower limb strength, four studies (18, 36, 38, 39) evaluated the peak torque and average power of the extensor muscles of the participants, and three of them (36, 38, 39) evaluated the total work of the extensor muscles. The synthesized data indicated that in the 60°/s isokinetic muscle strength of extensors, YJJ significantly enhanced extensor peak torque (*I*^2^ = 0%, *p* < 0.001, certainty of evidence = moderate), with a moderate improvement effect (Hedges’g = 0.47); extensor average power (*I*^2^ = 12.88%, *p* = 0.02, evidence certainty = moderate), with a small improvement effect (Hedges’g = 0.31); and extensor total work (*I*^2^ = 8.79%, *p* = 0.03, certainty of evidence = moderate), with a small improvement effect (Hedges’g = 0.29). However, in the 180°/s isokinetic muscle strength of extensors, YJJ only marginally improved extensor peak torque (*I*^2^ = 27.03%, *p* = 0.02, evidence certainty = moderate), with a small improvement effect (Hedges’g = 0.29); there was no significant improvement effect observed for extensor average power (*I*^2^ = 19.98%, *p* = 0.14, evidence certainty = moderate) (Hedges’g = 0.19) and extensor total work (*I*^2^ = 0%, *p* = 0.41, certainty of evidence = moderate) (Hedges’g = 0.11).

Three studies (18, 38, 39) assessed the peak torque and average power of the participants’ flexor muscles. The synthesized data showed that at 60°/s isokinetic muscle strength of the flexors, YJJ significantly increased flexor peak torque (*I*^2^ = 19.21%, *p* = 0.02, evidence certainty = moderate), with a moderate improvement effect (Hedges’g = 0.42); as well as flexor average power (*I*^2^ = 0%, *p* = 0.01, evidence certainty = moderate), with a moderate improvement effect (Hedges’g = 0.37). However, in the 180°/s isokinetic muscle strength of the flexors, YJJ did not demonstrate any improvement in flexor peak torque (*I*^2^ = 0%, *p* = 0.96, evidence certainty = moderate) (Hedges’g = 0.01) or flexor average power (*I*^2^ = 0%, *p* = 0.61, evidence certainty = moderate) (Hedges’g = −0.08).

#### 3.4.2 Physical performance in the older individuals

**Figure 4:**
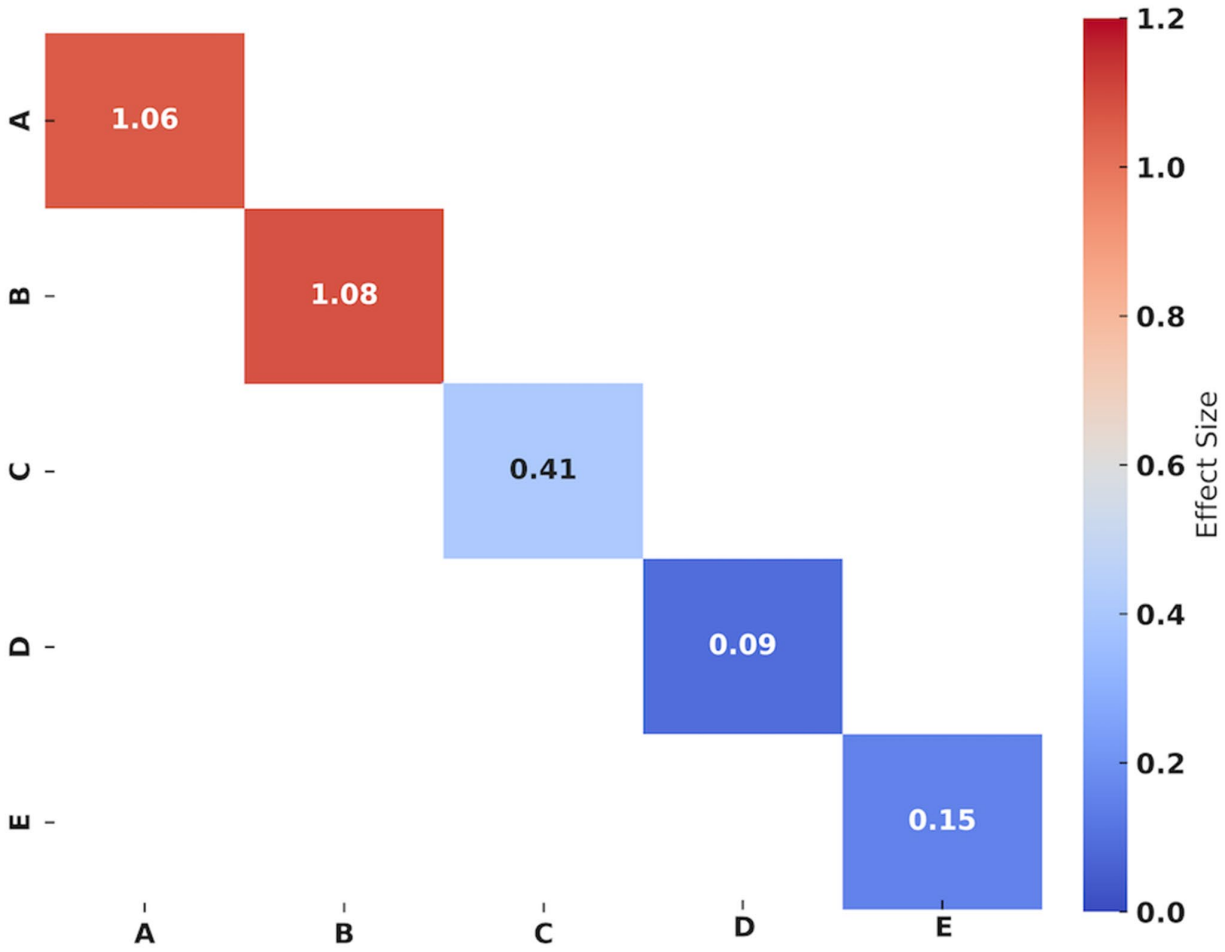
Effect size of Yi Jin Jing on physical performance in the older individuals. A: Chair sit-to-stand test; B: Squatting-to-standing test; C: Shoulder flexibility (left); D: Shoulder flexibility (right); E: Sit-and-reach test. Hedges’g values of 0.2–0.4 indicating small, 0.5–0.7 signifying medium, and ≥0.8 representing large effect sizes, respectively.

Table 3 and Figure 4 summarize the evidence of the effect of YJJ on physical performance in the older population. Forest plots and specific grade ratings are provided in Supplementary material.

**Table 3:** Summary of evidence on the impact of YJJ on physical performance in the older individuals.

Outcomes	Number of studies	Effect model	*I*^2^ (%)	Effect size (95%CI)	*p*-value	Certainty of evidence
Chair sit-to-stand test	3	Random model	69.30	2.45 (1.86, 3.04)	<0.001	⨁⨁◯◯ LOW
Squatting-to-standing test	3	Random model	90.91	2.38 (1.92, 2.85)	<0.001	⨁⨁◯◯ LOW
Shoulder flexibility (left)	2	Fixed model	0	1.33 (0.3, 2.35)	0.01	⨁⨁⨁◯ MODERATE
Shoulder flexibility (right)	2	Fixed model	0	0.29 (−0.78, 1.37)	0.06	⨁⨁⨁◯ MODERATE
Sit-and-reach test	3	Fixed model	0	1.20 (0.85, 3.25)	0.25	⨁⨁⨁◯ MODERATE

Three studies (15, 21, 37) assessed chair sit-to-stand test results in older individuals. The synthesized data indicated that the effect of YJJ on the chair sit-to-stand test was statistically significant (*I*^2^ = 69.30%, *p* < 0.001, certainty of evidence = low), with a large improvement effect (Hedges’g = 1.06). Sensitivity analysis showed that the heterogeneity of the chair sit-to-stand test decreased from 69.30 to 29.75% when one study was removed (15).

Three studies (15, 21, 37) reported the outcomes of the squatting-to-standing tests in older individuals. The synthetic data showed that the effect of YJJ on the chair sit-to-stand test was statistically significant (*I*^2^ = 90.91%, p < 0.001, certainty of evidence = low), with a large improvement effect (Hedges’g = 1.08). Sensitivity analysis revealed that the heterogeneity of the squatting-to-standing test decreased from 90.91 to 76.05% when one study was removed (15).

Two studies (21, 37) reported the outcomes of shoulder flexibility in older individuals. The synthetic data revealed that YJJ significantly increased left shoulder flexibility (*I*^2^ = 0%, *p* = 0.01, certainty of evidence = moderate), with a slight improvement effect (Hedges’g = 0.41); however, no effect was observed on right shoulder flexibility (*I*^2^ = 0%, *p* = 0.06, certainty of evidence = moderate) (Hedges’g = 0.09).

Three studies (21, 22, 37) investigated the outcomes of the sit-and-reach test in the older individuals. The synthetic data suggested that YJJ did not affect the sit-and-reach test (*I*^2^ = 0%, *p* = 0.25, the certainty of evidence = moderate) (Hedges’g = 0.15).

## 4 Discussion

This meta-analysis included 10 RCTs involving 590 patients. The overall risk of bias was assessed to be low. The methodological quality of these studies was generally moderate, and the quality of the main results ranging from low to moderate. The results of the meta-analysis showed that compared with the control group, YJJ significantly improved both muscle strength and physical performance in the older individuals.

Previous studies have demonstrated the beneficial effects of traditional Chinese exercises on muscle strength and overall physical function in older individuals (16, 17, 20). However, these studies have certain limitations. First, they often combined various traditional Chinese exercises without conducting subgroup analyses of the YJJ. For example, Wang et al. categorized exercise types into Tai Chi, Baduanjin, and other Qigong and found that traditional Chinese exercises significantly improved balance, physical performance, and muscle strength in older adults (24). Similarly, Wan et al.’s study, which included Tai Chi and Baduanjin, revealed that both exercises were beneficial for alleviating frailty and enhancing physical function in pre-frail and frail older adults, although Tai Chi did not improve handgrip strength in frail individuals (25). Additionally, two studies referenced sarcopenia, Niu et al. combined Tai Chi, Baduanjin, and YJJ, demonstrating the significant benefits of traditional Chinese exercises for lower limb muscle strength and physical function, but no improvement in handgrip strength (26). Guo et al.’s study on traditional Chinese medicine, including herbal medicine, acupuncture, and traditional Chinese exercises (including Tai Chi, Baduanjin, and YJJ), demonstrated the effectiveness of traditional Chinese medicine in treating sarcopenia as indicated by improvements in grip strength, chair stand test, timed up and go test, 6-m walking speed, and sit and reach (27). Second, these studies (24–27) did not calculate the effect size, which is a useful indicator in meta-analyses because it can statistically correct the variance caused by small sample sizes and synthesize data measured on different scales (28). Notably, a recent network meta-analysis reported that YJJ was the most effective method for enhancing balance and reducing their fear of falling in older individuals compared to Tai Chi, Baduanjin, and Wuqinxi (40). To the best of our knowledge, this study is the first to explore the specific effect sizes of YJJ on muscle strength and physical performance in an older population.

Although our study revealed a low heterogeneity in muscle strength, the results should be interpreted with caution because of the limited number of studies included. YJJ’s movement framework involves a series of complex motions, including rotation, bending, and extension of the forearm, as well as intricate finger and grip movements (41). This comprehensive exercise targets the muscles of the shoulders, arms, and hands, potentially improving handgrip strength and flexibility (12). Furthermore, YJJ plays a key role in knee joint stabilization through closed-chain exercises (17, 42, 43). It emphasizes isotonic contractions in lower-limb movements, particularly through extensive squatting, enhancing muscle coordination and strength around the joints (13). This meta-analysis suggests that YJJ substantially enhanced the peak torque, average power, and total work of both the extensor and flexor muscles during the 60°/s isokinetic muscle strength test. However, the gentle and progressive movement nature of YJJ may not be well suited for the 180°/s test, which depends on rapid strength and explosive muscular force. This indicates that YJJ is more appropriate for improving endurance and controlled strength than for high-speed activities.

This meta-analysis further demonstrated that YJJ exerted a significant positive effect on chair sit-to-stand and squatting-to-standing tests, which are key indices of lower limb strength and overall physical performance. The marked reduction in heterogeneity observed in these two tests likely stems from different participant selection criteria. Specifically, Zhu’s research focused on patients with sarcopenia diagnosed according to the AWGS criteria (11), whereas Wang and Jin’s study applied Roubenoff’s view to identify sarcopenia in individuals aged 60 and over (44). Therefore, YJJ can be considered an effective intervention to enhance lower limb strength and physical performance in the older individuals.

Aging is associated with decreased skeletal muscle quality and functionality, leading to diminished joint mobility and muscle flexibility (45). This deterioration affects the performance of older individuals in flexibility assessments, such as sit-and-reach performance (46). Conversely, YJJ, which includes arm rotations and lifts, may improve the range of motion and coordination of the shoulder joint, potentially leading to increased shoulder flexibility. Studies have shown that asymmetric use of the dominant hand in daily activities affects muscle and joint functions (47). Considering that the left shoulder is less involved in high-intensity activities in daily life, it may be more responsive to YJJ, resulting in a significant improvement in flexibility.

## 5 Conclusion

In summary, our findings indicate that YJJ significantly enhances specific aspects of physical performance, notably the chair sit-to-stand test and squatting-to-standing test. Additionally, moderate improvements were observed in handgrip strength and various measures of knee flexor and extensor strength. In contrast, YJJ had no significant effects on certain metrics, including extensor and flexor average power at 180°/s, extensor total work at 180°/s, flexor peak torque at 180°/s, right shoulder flexibility, and the sit-and-reach test.

## 6 Limitations and directions for future research

This study has several limitations. First, it did not include high-quality studies, which may have affected the robustness of our conclusions. Second, the reliance on expert opinions for sarcopenia diagnosis in earlier studies may introduce a potential risk of bias and uncertainty. Third, the limited number of eligible studies and their small sample sizes are constraints. Future studies should adopt rigorous research designs, including clear selection criteria, sample size calculations, randomization, allocation concealment, and blinding. Additionally, it is crucial to adjust exercise parameters, such as frequency, duration, and intensity, to match the physiological characteristics of the older individuals and meticulously record any adverse events and reasons for dropout. Importantly, our findings revealed that Yi Jin Jing had a small but significant effect on handgrip strength in older individuals, suggesting that combining it with resistance exercises could achieve better outcomes.

## Data Availability

Publicly available datasets were analyzed in this study. This data can be found here: the data presented in this study are available on reasonable request from the corresponding author.

## Footnotes

^1^https://www.crd.york.ac.uk/PROSPERO/
