# Supplementary material for: Effects of Yi Jin Jing on enhancing muscle strength and physical performance in older individuals: a systematic review and meta-analysis
Source: Front Med (Lausanne). 2024 Oct 25;11:1441858. doi: 10.3389/fmed.2024.1441858 (PMC11543491; doi:10.3389/fmed.2024.1441858)
Supplement: Supplementary file 1 [file Data_Sheet_1.docx]

Supplementary Material

**
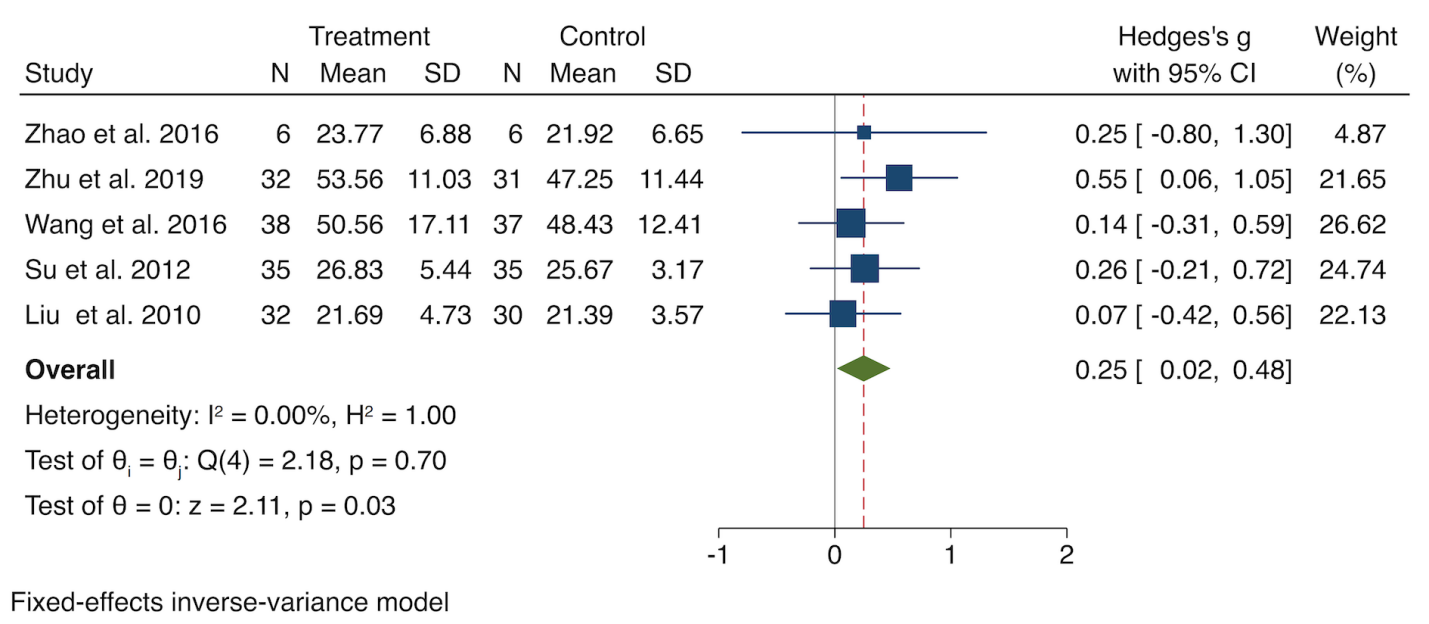
**

**Supplementary Figure 1**. Forest plot of handgrip strength.

**
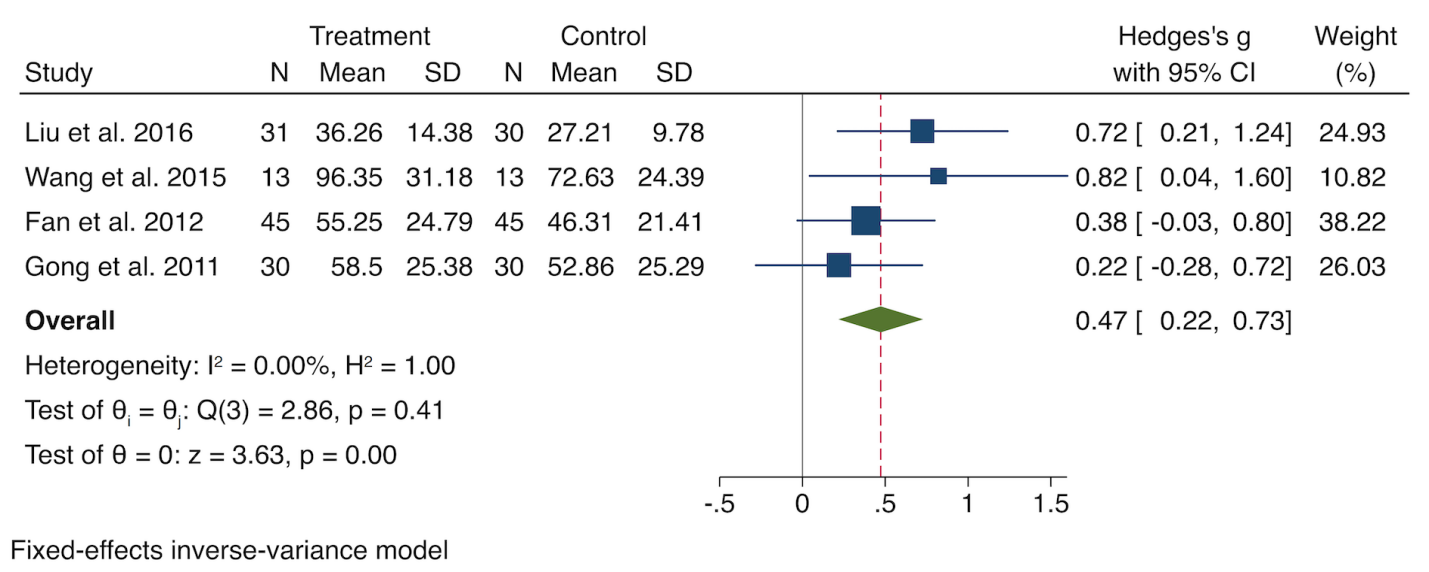
**

**Supplementary Figure 2**. Forest plot of isokinetic muscle strength (60°/s extensor’ PT).

**
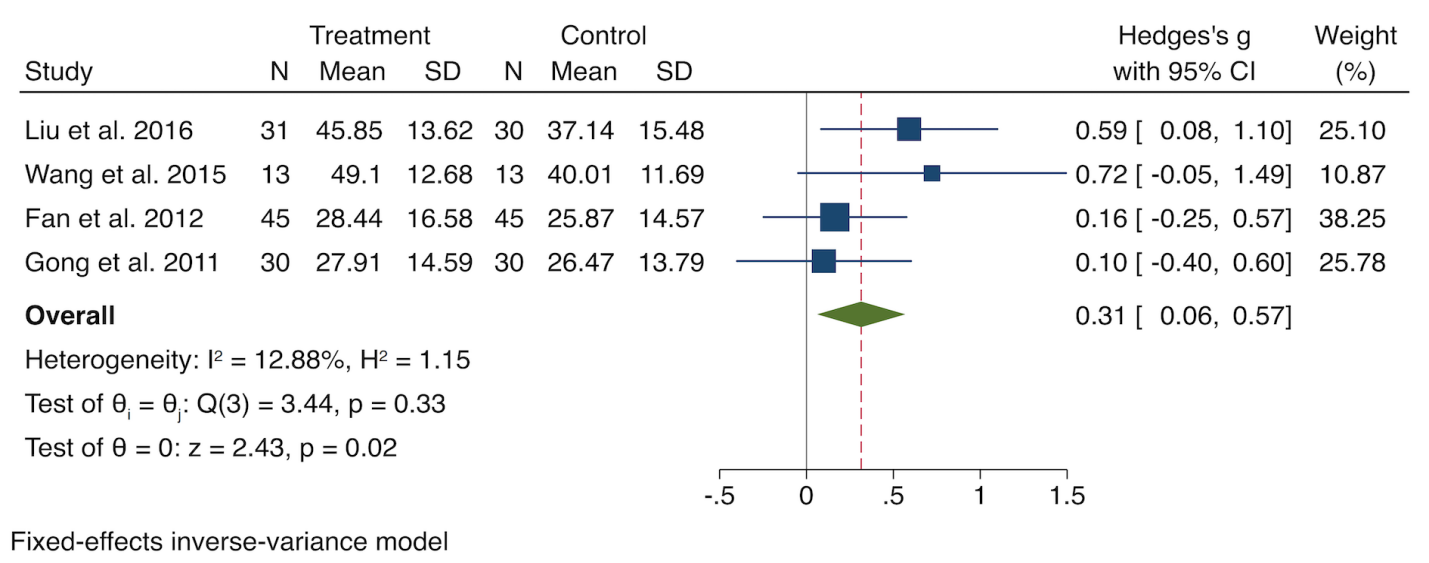
**

**Supplementary Figure 3**. Forest plot of isokinetic muscle strength (60°/s extensor’ AP).

**
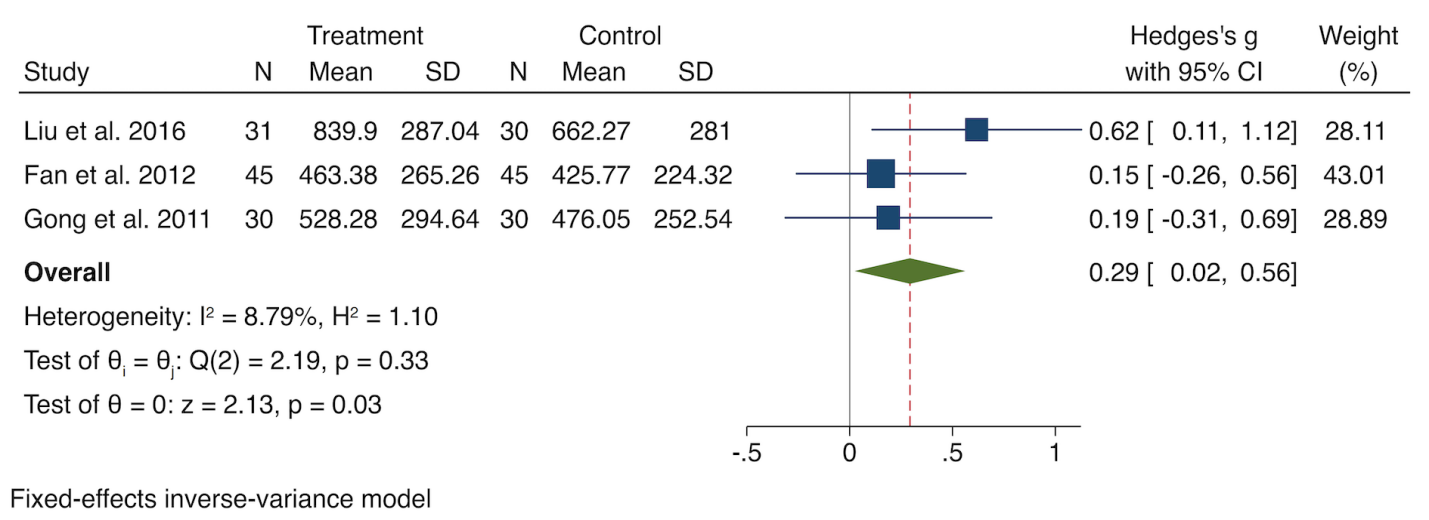
**

**Supplementary Figure 4**. Forest plot of isokinetic muscle strength (60°/s extensor’ TW).

**
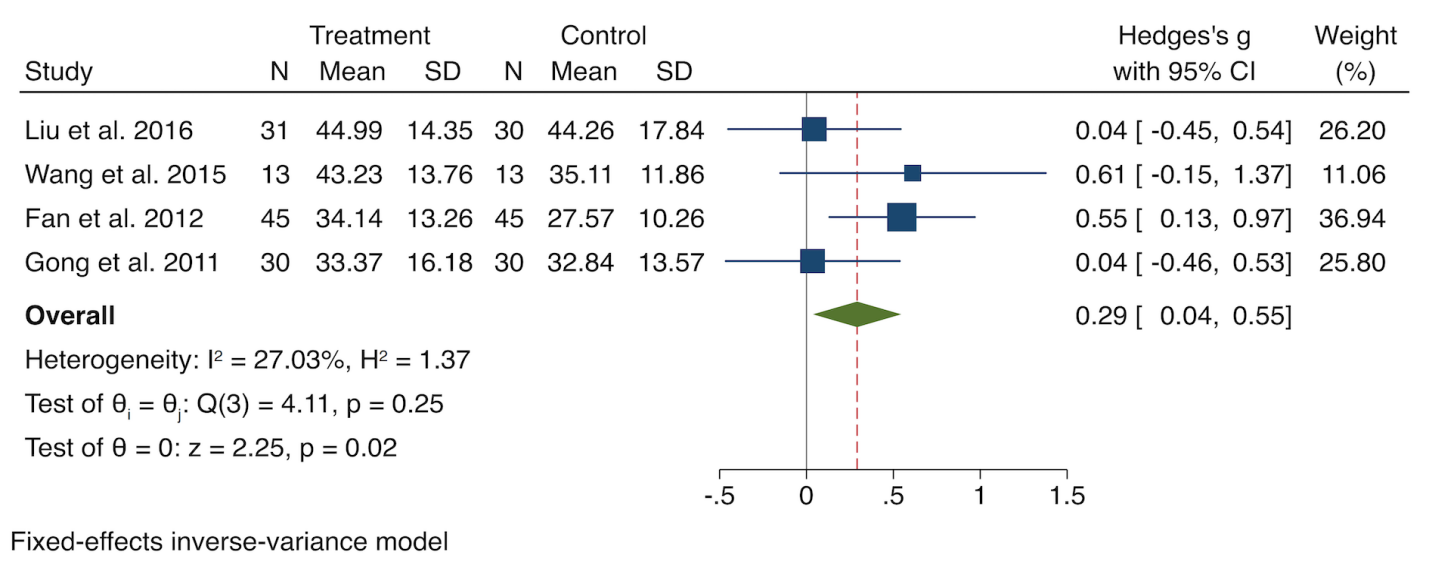
**

**Supplementary Figure 5**. Forest plot of isokinetic muscle strength (180°/s extensor’ PT).


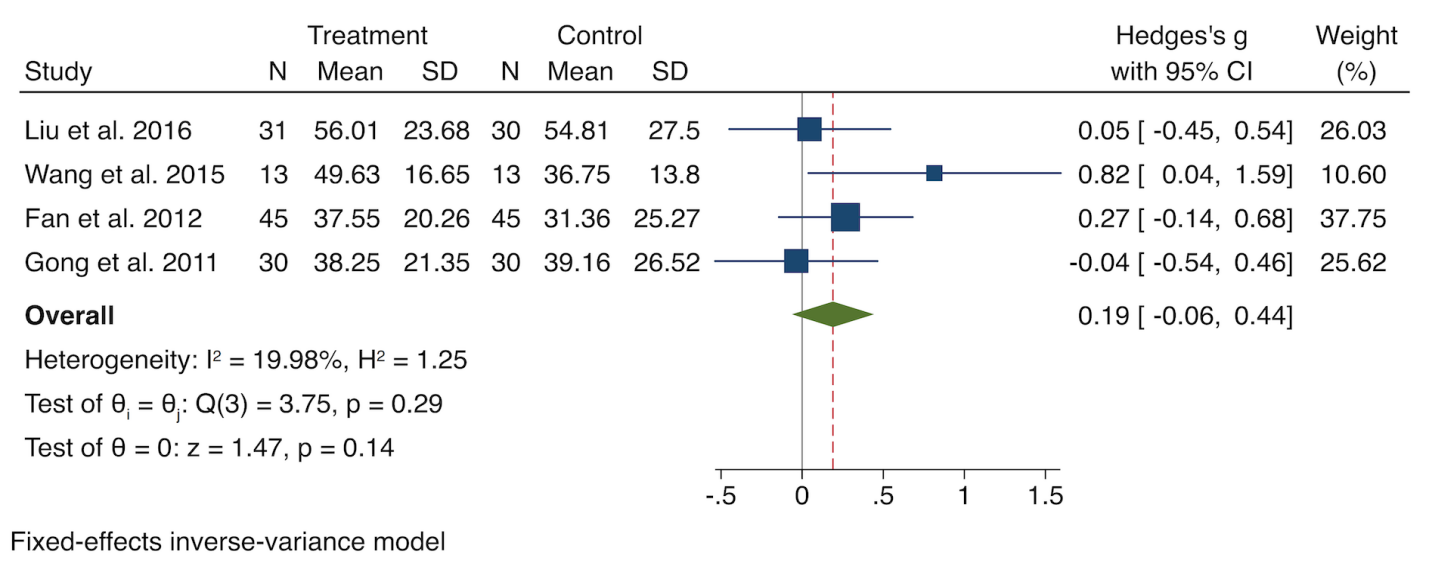


**Supplementary Figure 6**. Forest plot of isokinetic muscle strength (180°/s extensor’ AP).

**
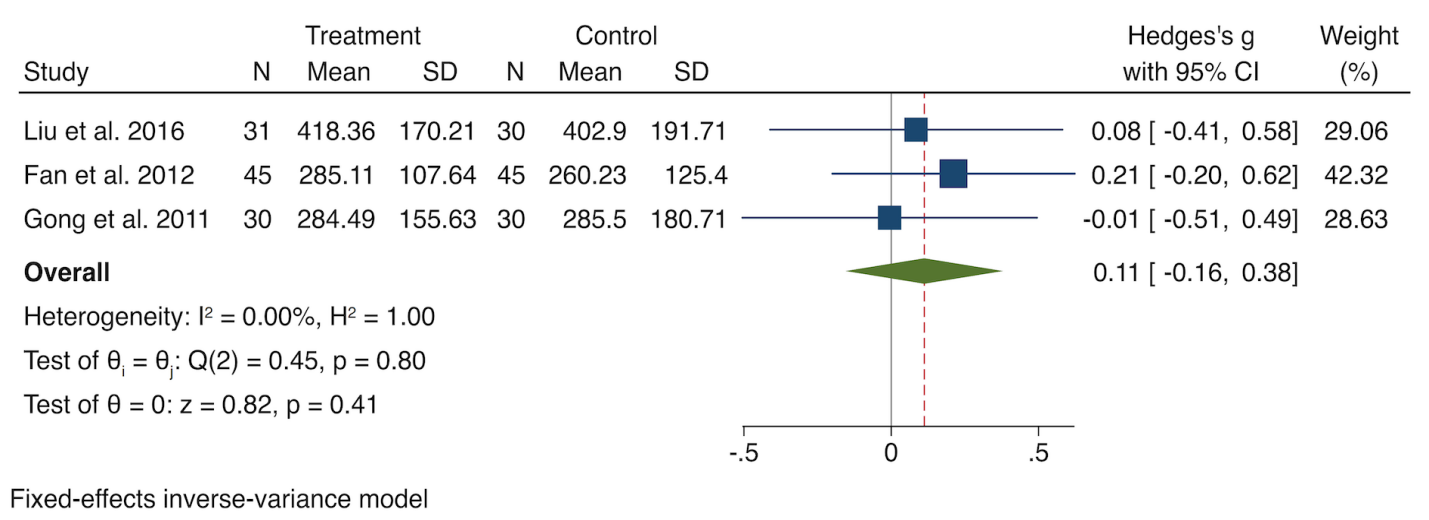
**

**Supplementary Figure 7**. Forest plot of isokinetic muscle strength (180°/s extensor’ TW).


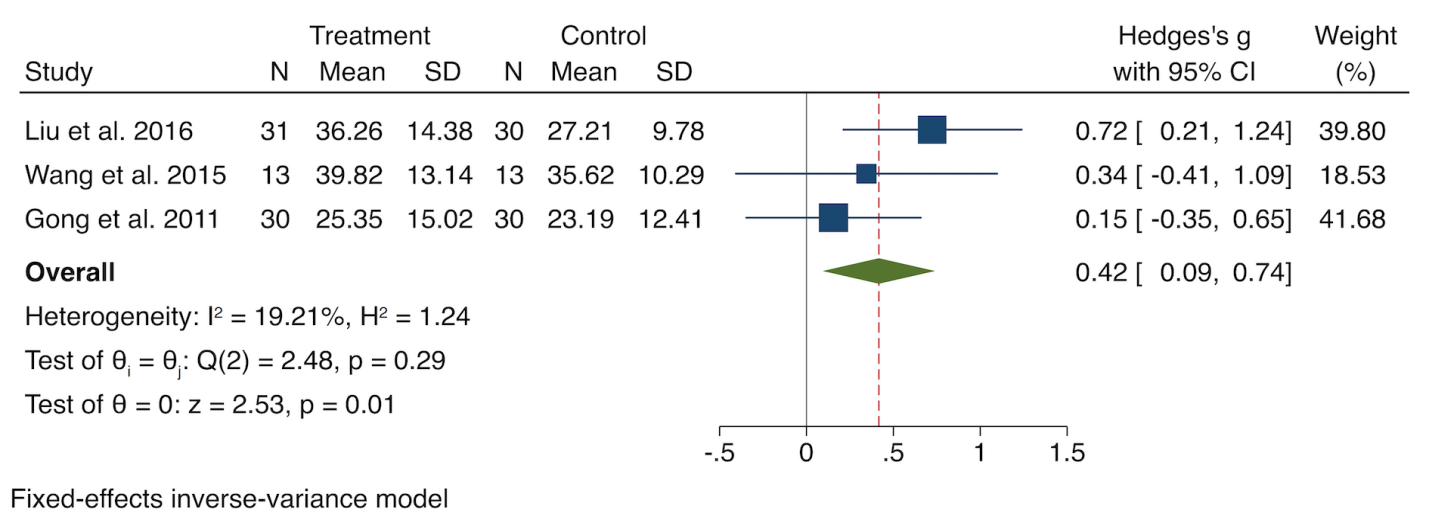


**Supplementary Figure 8**. Forest plot of isokinetic muscle strength (60°/s flexor’ PT).


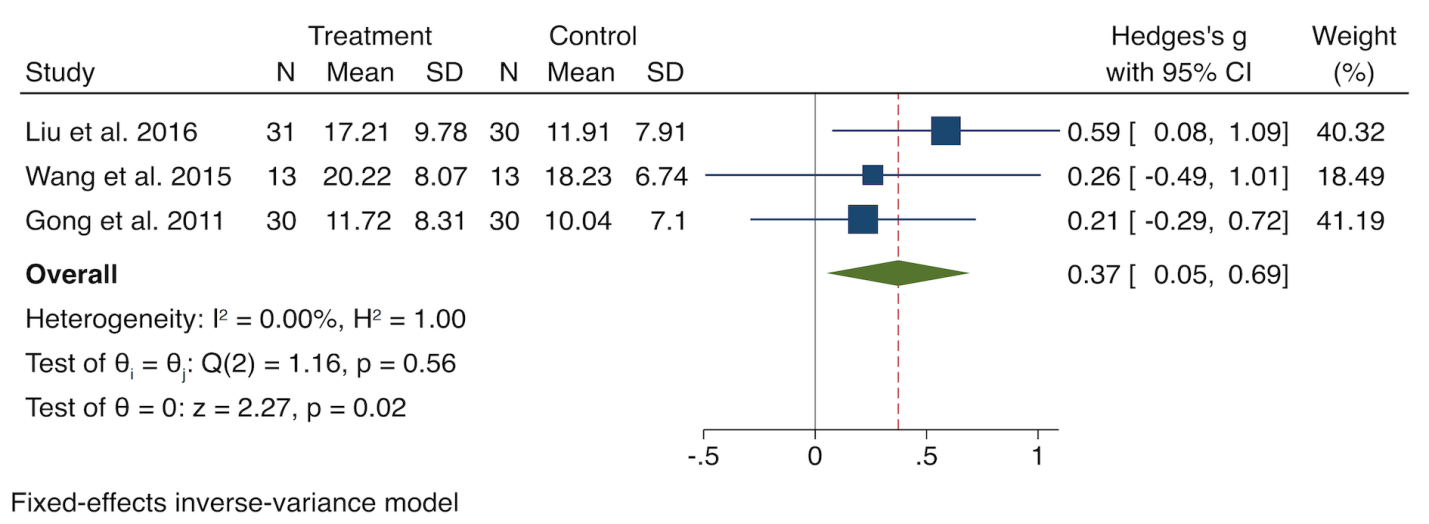


**Supplementary Figure 9**. Forest plot of isokinetic muscle strength (60°/s flexor’ AP).


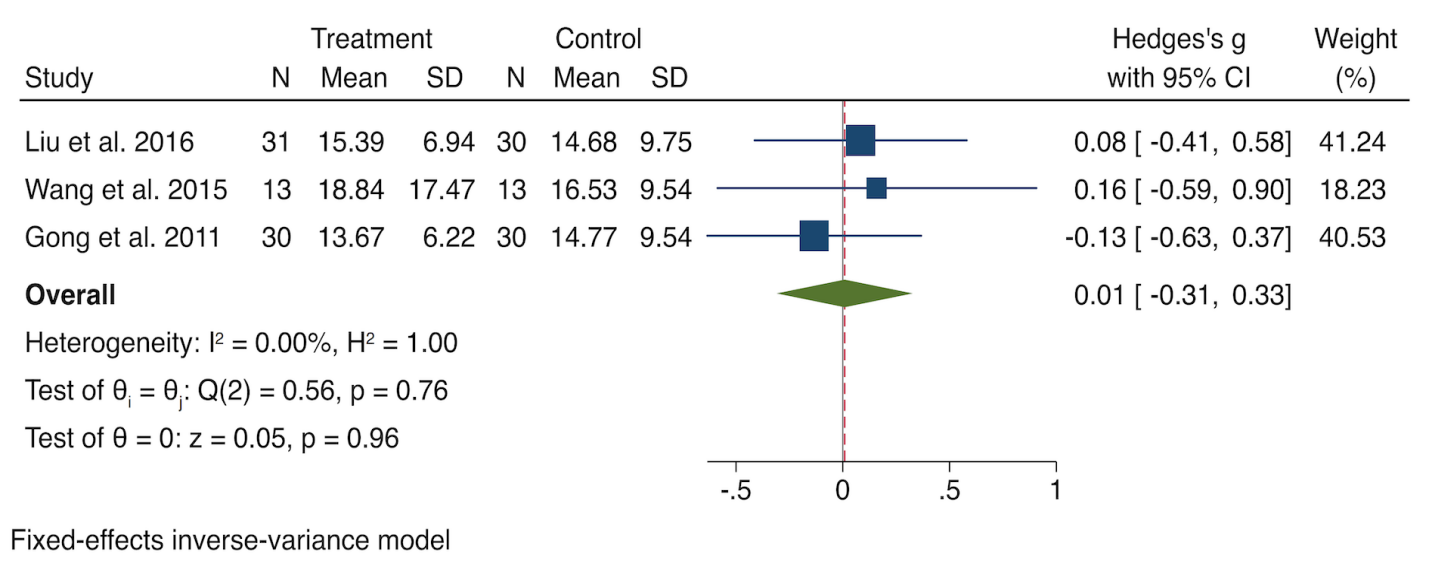


**Supplementary Figure 10**. Forest plot of isokinetic muscle strength (180°/s flexor’ PT).


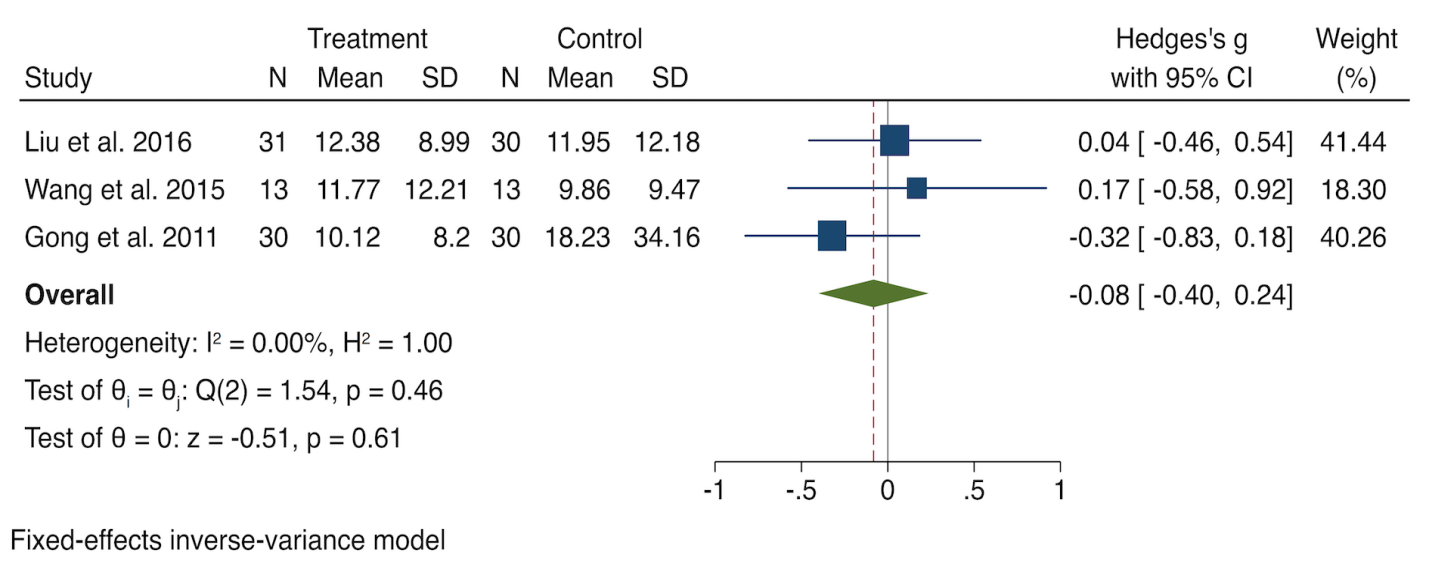


**Supplementary Figure 11**. Forest plot of isokinetic muscle strength (180°/s flexor’ AP).


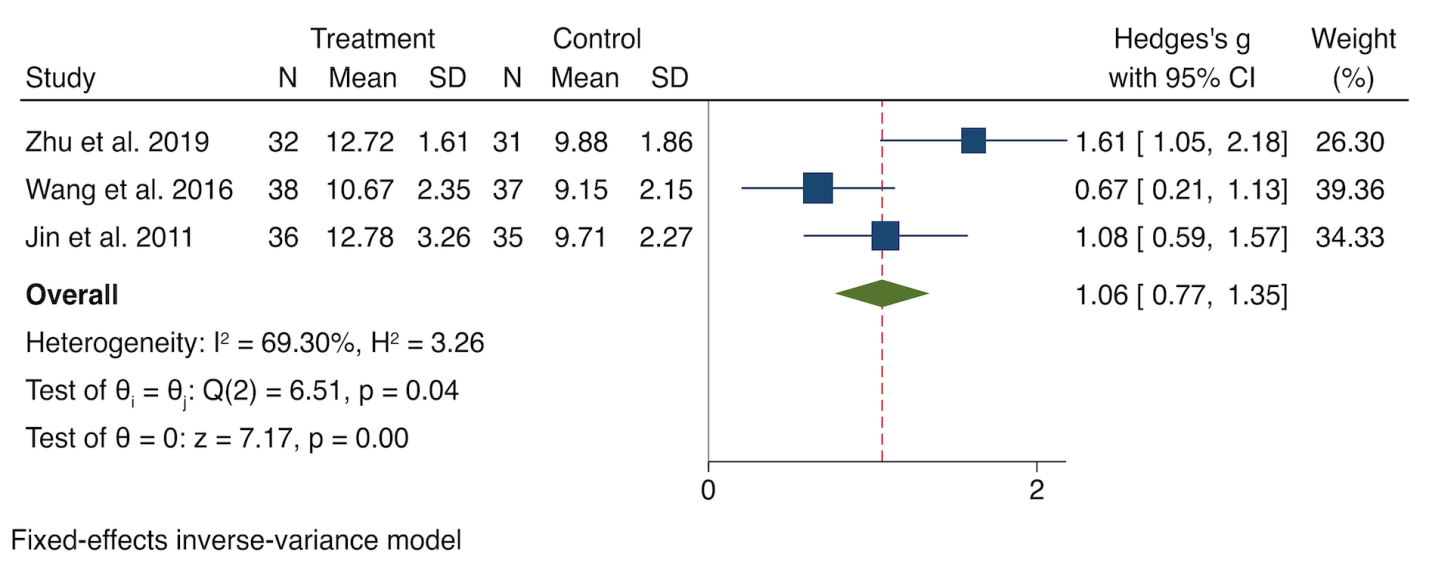


**Supplementary Figure 12**. Forest plot of chair sit-to-stand test.


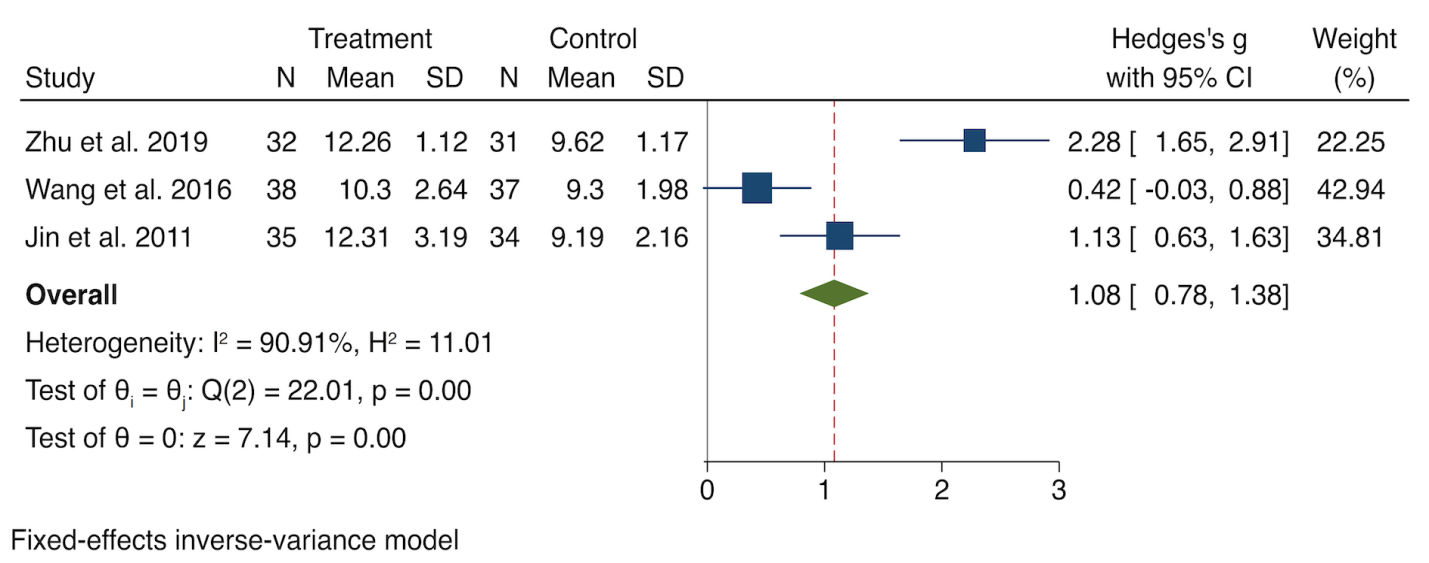


**Supplementary Figure 13**. Forest plot of squatting-to-standing test.


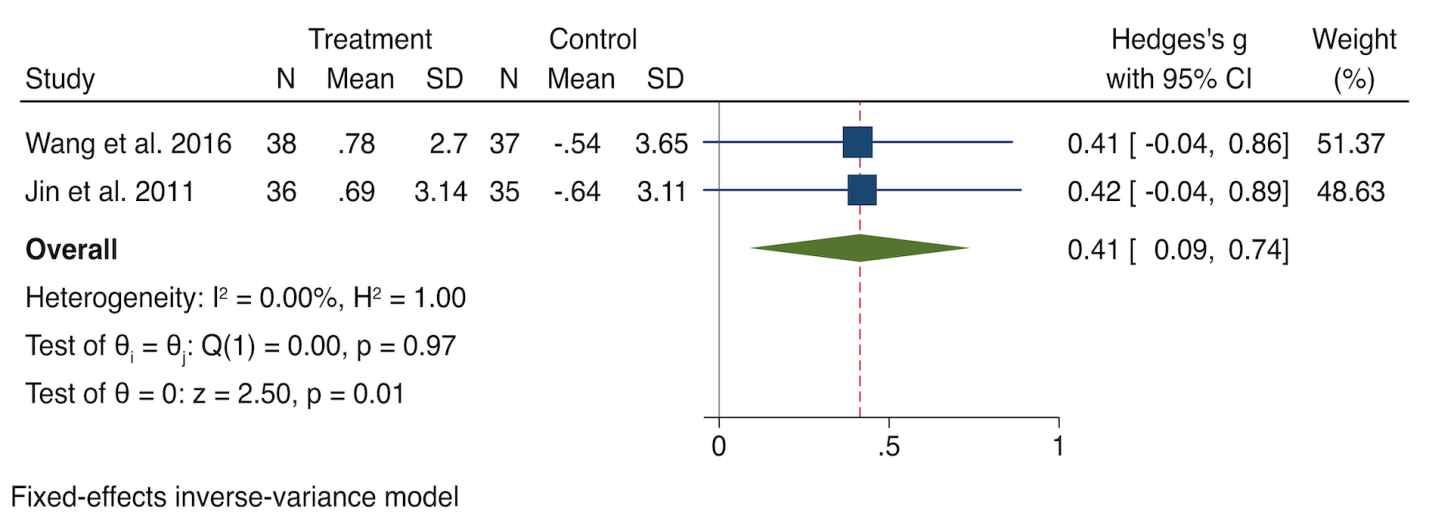


**Supplementary Figure 14**. Forest plot of left shoulder flexibility.


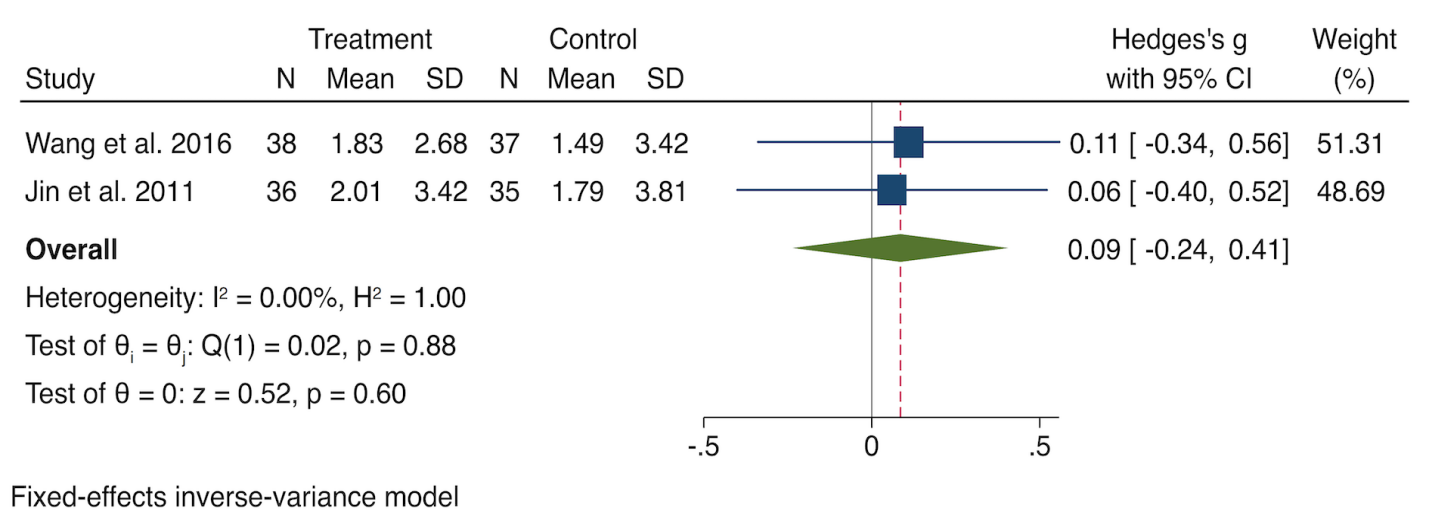


**Supplementary Figure 15**. Forest plot of right shoulder flexibility.


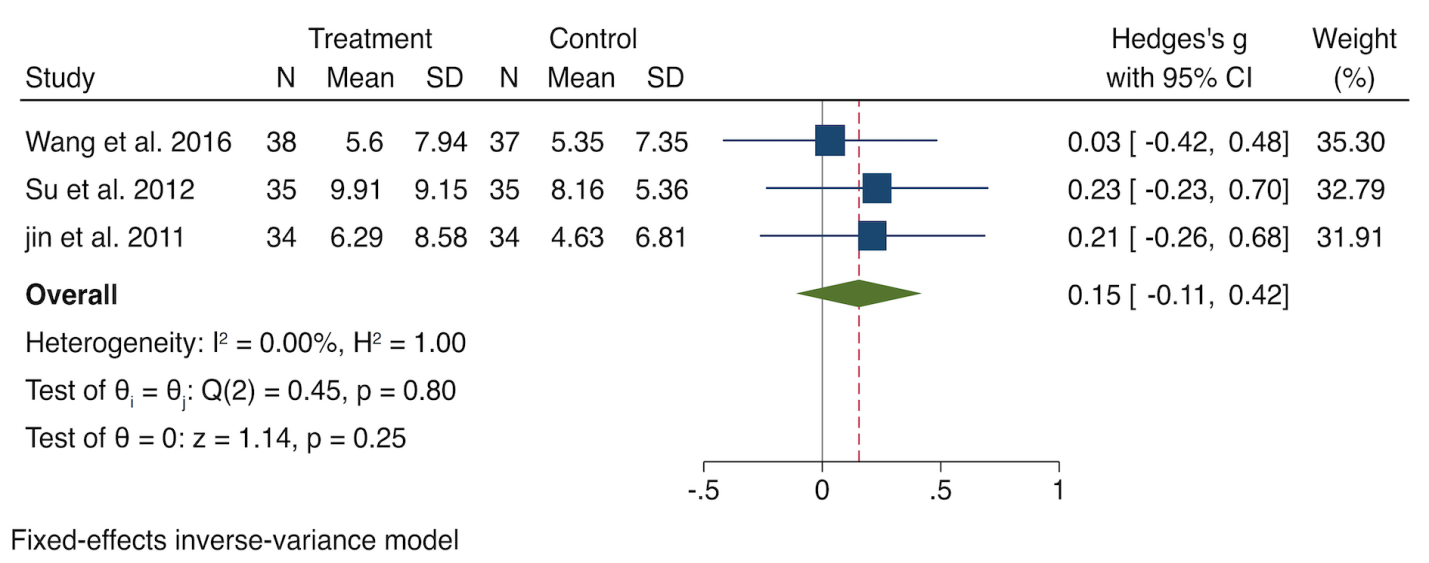


**Supplementary Figure 16**. Forest plot of sit-and-reach test.

Note. A: 60°/s extensor’ PT; B: 60°/s fextensor’ AP; C: 60°/s extensor’ TW
