# Supplementary material for: Effects of Yi Jin Jing on enhancing muscle strength and physical performance in older individuals: a systematic review and meta-analysis
Source: Front Med (Lausanne). 2024 Oct 25;11:1441858. doi: 10.3389/fmed.2024.1441858 (PMC11543491; doi:10.3389/fmed.2024.1441858)
Supplement: Supplementary file 2 [file Table_1.docx]

Supplementary Material

**Supplementary Table 1.** Grade ratings of muscle strength and physical performance in the older individuals.

| **Outcomes** | **Number**  **of studies** | **Quality assessment** | | | | | **Number of Participants** | | **Effect** | **Quality** | **Importance** |
| --- | --- | --- | --- | --- | --- | --- | --- | --- | --- | --- | --- |
|  |  | **Risk of bias** | **Inconsistency** | **Indirectness** | **Imprecision** | **Publication bias** | **Trail**  **group** | **Control**  **group** | **MD (95%Cl)** |  |  |
| **Muscle strength** | | | | | | | | | | | |
| Handgrip strength | 5 | Serious^a^ | No serious | No serious | Serious^c^ | Undetected | 143 | 139 | 1.18 higher (0.22 lower to 2.58 higher) | ⨁⨁◯◯  LOW^ac^ | IMPORTANT |
| 60°/s extensor’ PT | 4 | Serious^a^ | No serious | No serious | No serious | Undetected | 119 | 118 | 9.26 higher  (4.58 higher to 13.95 higher) | ⨁⨁⨁◯  MODERATE^a^ | IMPORTANT |
| 60°/s extensor’ AP | 4 | Serious^a^ | No serious | No serious | No serious | Undetected | 119 | 118 | 4.48 higher  (1.15 higher to 8.53 higher) | ⨁⨁⨁◯  MODERATE^a^ | IMPORTANT |
| 60°/s extensor’ TW | 3 | Serious^a^ | No serious | No serious | No serious | Undetected | 106 | 105 | 76.21 higher  (5.17 higher to 147.26 higher) | ⨁⨁⨁◯  MODERATE^a^ | IMPORTANT |
| 180°/s extensor’ PT | 4 | Serious^a^ | No serious | No serious | No serious | Undetected | 119 | 118 | 4.46 higher  (1.02 higher to 7.90 higher) | ⨁⨁⨁◯  MODERATE^a^ | IMPORTANT |
| 180°/s extensor’ AP | 4 | Serious^a^ | No serious | No serious | No serious | Undetected | 119 | 118 | 5.25 higher  (0.42 higher to 10.91 higher) | ⨁⨁⨁◯  MODERATE^a^ | IMPORTANT |
| 180°/s extensor’ TW | 3 | Serious^a^ | No serious | No serious | No serious | Undetected | 106 | 105 | 18.05 higher  (20.11 higher to 56.21 higher) | ⨁⨁⨁◯  MODERATE^a^ | IMPORTANT |
| 60°/s flexor’ PT | 3 | Serious^a^ | No serious | No serious | No serious | Undetected | 74 | 73 | 5.56 higher  (1.54 higher to 9.77 higher) | ⨁⨁⨁◯  MODERATE^a^ | IMPORTANT |
| 60°/s flexor’ AP | 3 | Serious^a^ | No serious | No serious | No serious | Undetected | 74 | 73 | 2.99 higher  (0.38 higher to 5.60 higher) | ⨁⨁⨁◯  MODERATE^a^ | IMPORTANT |
| 180°/s flexor’ PT | 3 | Serious^a^ | No serious | No serious | No serious | Undetected | 74 | 73 | 0.06 lower  (0.29 lower to 2.78 higher) | ⨁⨁⨁◯  MODERATE^a^ | IMPORTANT |
| 180°/s flexor’ AP | 3 | Serious^a^ | No serious | No serious | No serious | Undetected | 74 | 73 | 0.17 lower  (4.44 lower to 4.09 higher) | ⨁⨁⨁◯  MODERATE^a^ | IMPORTANT |
| **Physical activity ability** | | | | | | | | | | | |
| Chair sit-to-stand test | 3 | Serious^a^ | Serious^b^ | No serious | No serious | Undetected | 105 | 102 | 2.45 higher  (1.86 higher to 3.04 higher) | ⨁⨁◯◯  LOW^ab^ | IMPORTANT |
| Squatting-to-standing test | 3 | Serious^a^ | Serious^b^ | No serious | No serious | Undetected | 105 | 102 | 2.38 higher  (1.92 higher to 2.85 higher) | ⨁⨁◯◯  LOW^ab^ | IMPORTANT |
| Shoulder flexibility  (left) | 2 | Serious^a^ | No serious | No serious | No serious | Undetected | 74 | 72 | 1.33 higher  (0.3 higher to 2.35 higher) | ⨁⨁⨁◯  MODERATE^a^ | IMPORTANT |
| Shoulder flexibility (right) | 2 | Serious^a^ | No serious | No serious | No serious | Undetected | 74 | 72 | 0.29 higher  (0.78 lower to 1.37higher) | ⨁⨁⨁◯  MODERATE^a^ | IMPORTANT |
| Sit-and-reach test | 3 | Serious^a^ | No serious | No serious | No serious | Undetected | 107 | 106 | 1.20 higher  (0.85 higher to 3.25 higher) | ⨁⨁⨁◯  MODERATE^a^ | IMPORTANT |

|  |
| --- |

|  |
| --- |

^a^ Unclear blindness or unclear allocation concealment

^b^ Significant heterogeneity

^c^ Small sample size
